# Case-mix Adjustment of Patient Reported Experience Measures (PREMs): a rapid review to inform benchmarking practices across inpatient health centers in Switzerland

**DOI:** 10.1186/s41687-025-00922-0

**Published:** 2025-07-15

**Authors:** Katrina Obas, Chiara Storari, Francesca Giuliani

**Affiliations:** 1 Universitätsspital Zürich, Qualitätsmanagement & Patientensicherheit, Rämistrasse 100, Zürich, 8091 Switzerland; 2 Unisanté, University Center for Primary Care and Public Health, Department of Epidemiology and Health Systems, University of Lausanne, Lausanne, Switzerland

**Keywords:** Patient Reported Experience Measures, Quality improvement, Case-mix adjustment, Switzerland

## Abstract

**Aim:**

The rapid review aimed to analyse current practices and recommendations regarding case-mix adjustment for benchmarking Patient Reported Experience Measures (PREMs) across inpatient health centres. Findings will inform the applicability of case-mix adjustment to PREMs in the Swiss context.

**Methods:**

We searched PubMed, Embase, and Web of Science for studies which met the following criteria: PREMs is a main outcome, study from a European country with a national inpatient PREMs survey, study with adult patients in acute care setting, and evaluates the effect of case-mix adjustment on PREMs. Screening and appraisal were performed by an experienced epidemiologist. A narrative evidence synthesis was undertaken to address the review question, with support of tables to summarize evidence on case-mix variables and statistical methods.

**Results:**

Seven studies (n = 301,833) were included. All supported case-mix adjustment to some extent, though variables used for case mix varied, complicating standardization. Concerns included the risk of masking quality differences. To address this, several authors advocated reporting both adjusted and unadjusted scores. Only one study included language spoken as a case mix variable—a key factor in Switzerland.

**Discussion:**

Case-mix adjustment can enhance fairness in PREM-based benchmarking but must be applied cautiously. For multilingual contexts like Switzerland, local relevance of adjustment variables should be evaluated. A stepwise, transparent approach is recommended to avoid obscuring true performance differences.

## Introduction

### Patient experience as a key indicator of care quality

In the dynamic landscape of healthcare, the pursuit of excellence in patient care has evolved beyond traditional clinical outcomes. Since recognizing the importance of the patient perspective in evaluating healthcare quality [1], there has been a notable shift towards incorporating Patient Reported Experience Measures (PREMs) as essential indicators in hospital assessments. This paradigmatic change from a paternalistic model reflects a growing acknowledgment that the patient experience is not merely a subjective element, but a critical determinant of overall healthcare quality. As hospitals strive to achieve patient-centered care, the integration of PREMs into quality assessments has gained prominence as a powerful tool to capture and respond to the multifaceted dimensions of the patient experience [2]. Striving towards continuous improvement of health services, PREMs can be used as tools for benchmarks across hospitals and their units [2].

### Challenges in fair benchmarking

The use of PREMs to evaluate the quality of care come with inherent challenges. The rating of healthcare experience is influenced not only by the quality of care provided to patients, but also by ‘exogenous’ factors, such as the sociodemographic characteristics of patients and the severity of their illness. Judgments about the quality of care may therefore be influenced by differential response tendencies between these patient groups. A review of the literature [3] found that younger and sicker patients tended to rate their experience more critically compared to their counterparts, while gender was associated with rating tendencies, but differed across healthcare aspects. This puts hospitals with a higher proportion of poorer-rating demographics at a disadvantage in PREMs reporting when compared to a hospital providing the same quality of care but with a more favourably-rating patient group or “case mix”. Case mix refers to the grouping of patients based on shared characteristics like demographic or health status, often used in healthcare to classify and analyze the diversity, complexity, and resource needs of patient populations.

One way to address differences in rating tendencies is through statistical methods such as case-mix adjustment. Commonly used in healthcare, case-mix adjustment accounts for variations in patient populations when comparing outcomes or performance across providers or systems. It involves adjusting observed hospital performance scores to reflect differences in patient characteristics that may affect outcomes or experiences. Techniques like multilevel analysis help isolate the influence of these factors, creating a fairer basis for comparison. By controlling for variables outside a provider’s control, case-mix adjustment ensures more accurate and equitable PREMs analyses [4].

Recognizing that patient populations vary widely in terms of demographic factors and health conditions, case mix adjustment allows for a more nuanced interpretation of PREMs data. By accounting for these diverse patient characteristics, healthcare providers can obtain a clearer understanding of the genuine impact of their services on the patient experience. This adjustment enables fairer comparisons between different healthcare institutions, as it helps mitigate the influence of patient-specific factors beyond the influence of healthcare that may affect reported experiences. In essence, the importance of case-mix adjustment in PREMs analysis lies in its ability to enhance the validity and reliability of the data, thereby facilitating more informed decisions for the advancement of overall healthcare quality. However, there is no consensus to date on a standardized method for case mix adjustment for the use of PREMs in benchmarking health centers. A major challenge of case mix adjustment is the risk of adjusting away actual differences in quality. The methodology used in benchmarking are currently highly criticized in this respect.

### Specific relevance to Switzerland

**Fig. 1:**
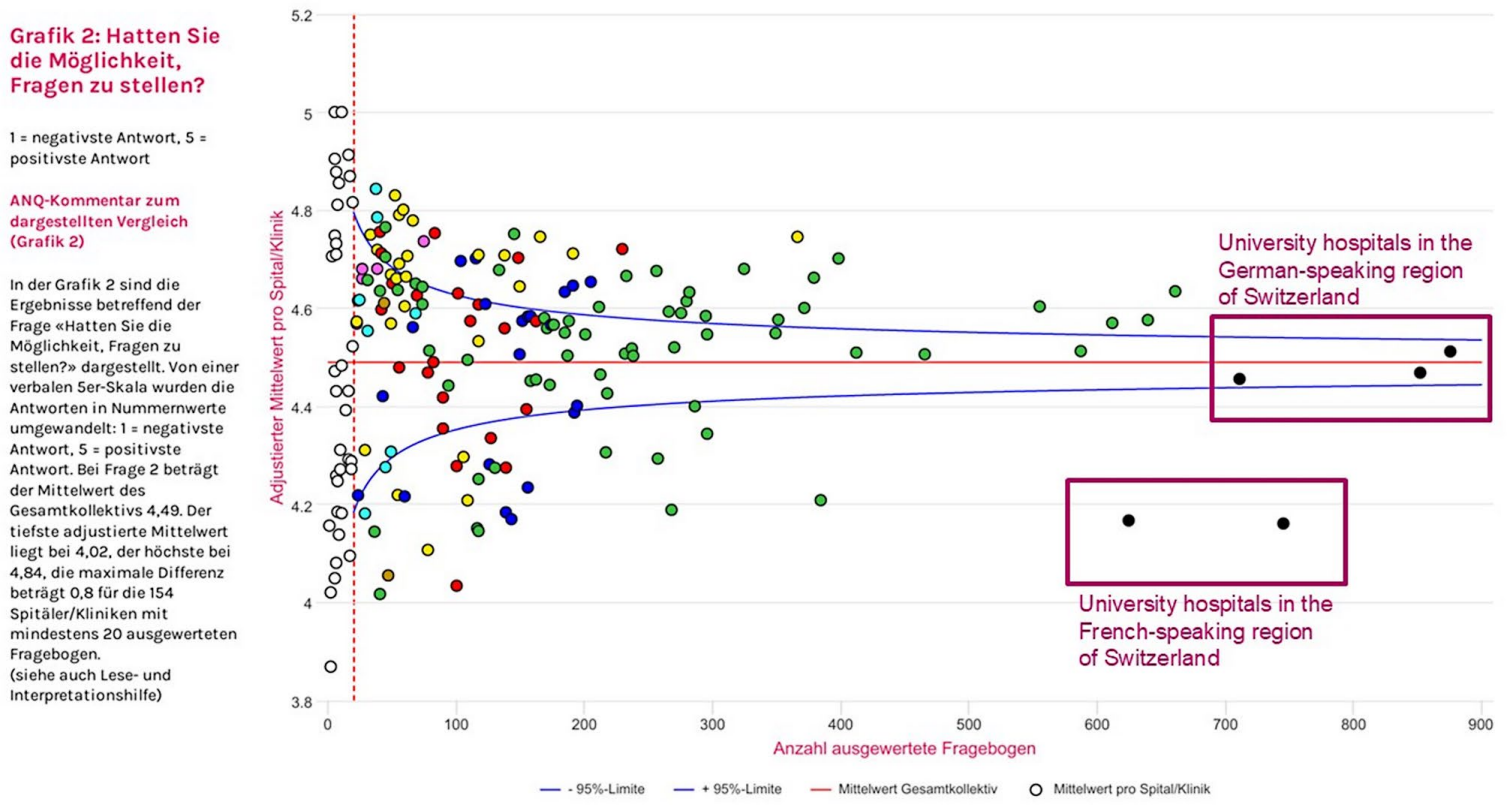
Inpatient rating on the “possibility to ask questions” from swiss national PREMs survey in 2023, Swiss National Association for Quality Development in Hospitals and Clinics [6]. Rating from 1 (most negative response) to 5 (most positive response) on the x axis. Number of respondents per hospital on the y axis

The Swiss National Association for Quality Development in Hospitals and Clinics (ANQ) currently coordinates the measurement and benchmarking of PREMs across all Swiss acute (inpatient) hospitals. PREMs are measured every two years using 6 questions. Until present, benchmarking has been conducted without case mix adjustment. Comparing PREMs across Switzerland however is particularly challenging due to the nation’s cultural diversity, with four linguistic regions, including French, Romansch, Italian and German Speaking regions. These cultural differences may translate to rating tendencies. For example, rating tendencies in one study differed across six European countries (Germany, Greece, Italy, Spain, France and the United Kingdom), and was attributed to differing cultural values [5]. Ethnicity as a case mix may mask poor poorer care among minorities where health inequity exists. Although diverse in cultural values and history, Switzerland is rather homogeneous in case-mix compared to settings with more diversity like the USA. Nevertheless, consistent differences in PREM ratings in Switzerland conducted by the ANQ have been observed over the last 10 years between University Hospitals from French Speaking and German Speaking regions (Fig. 1) [6].

The question remains whether observed differences are related to exogenous factors or differences in healthcare quality. Direct comparisons are flawed in the inpatient Swiss context, emphasizing the need for evidence-based approaches that consider each hospital’s unique characteristics and the diverse populations they serve. Achieving meaningful cross-hospital comparisons in this context requires addressing population disparities. Understanding how other countries have addressed these rating differences among inpatient settings would inform decisions about how to apply it in the Swiss context.

This Rapid Review aims to synthesize existing evidence on case-mix adjustment practices for comparing PREMs across inpatient health centers in similar contexts as Switzerland to inform fair benchmarking and national-level decision-making in Switzerland. To our knowledge, no such review has yet been conducted. By identifying methodologies and best practices, the review seeks to guide the development of evidence-based statistical methods and reporting, ensuring accurate and fair assessment of patient experiences in Switzerland.

## Methods

This Rapid Review serves as an accelerated systematic evidence synthesis on case-mix adjustment of Patient Reported Experience Measures in the comparison between inpatient health centres. The review followed the methodology outlined by [7], and was made rapid by limiting the search to three databases, including English only publications, limiting geographic location of studies, excluding grey literature and by formulating a targeted search question.

### Literature search

Electronic searches were conducted in three databases (PubMed, Embase, Web of Science]. The search strategy used key terms related to (a) Patient Reported Experience Measures, (b) Case Mix Adjustment, (c) studies from a similar context– therefore European countries which also regularly conduct a national PREMs survey (Austria, Czech Republic, Denmark, France, Germany, Ireland, Luxembourg, Netherlands, Norway, Poland, Sweden, Switzerland and the United Kingdom [8] and (d) Comparison or Benchmarking. Terms were combined with MeSH terms and the Boolean operators ‘AND’ and ‘OR’. The full search terms are included in the appendix.

#### Inclusion and exclusion criteria

Inclusion criteria were (a) studies with datasets from European countries which are reported to have a national PREM survey (Austria, Czech Republic, Denmark, France, Germany, Ireland, Luxembourg, Netherlands, Norway, Poland, Sweden, Switzerland and the United Kingdom [8], (b) studies assessing the effect of case-mix adjustment on PREMs with the aim to compare different health centres.

Exclusion criteria include: (a) the comparison of non-acute health centers such as primary care, (b) did not include PREMs as an outcome (for example only looking at Patient Reported Outcome Measures), (c) did not describe methods of case-mix adjustment, (d) dataset not from one of the countries of interest, (e) patients were younger than 18 years of age.

#### Screening protocol

The screening and appraisal of articles was conducted by KO, an epidemiologist with a doctoral degree and clinical experience with patients.

#### Data items and data abstraction process

A data abstraction form was determined a priori. Data items included study characteristics (for example, first author, year of publication, country), case-mix variables included in the study, PREMs used, statistical methods used for case mix adjustment and for evaluating the impact of case mix adjustment on PREM scores).

#### Synthesis

A narrative evidence synthesis was undertaken to address the review question, with support of tables to summarize evidence on case-mix variables and statistical methods.

## Results

### Studies included in review

**Fig. 2:**
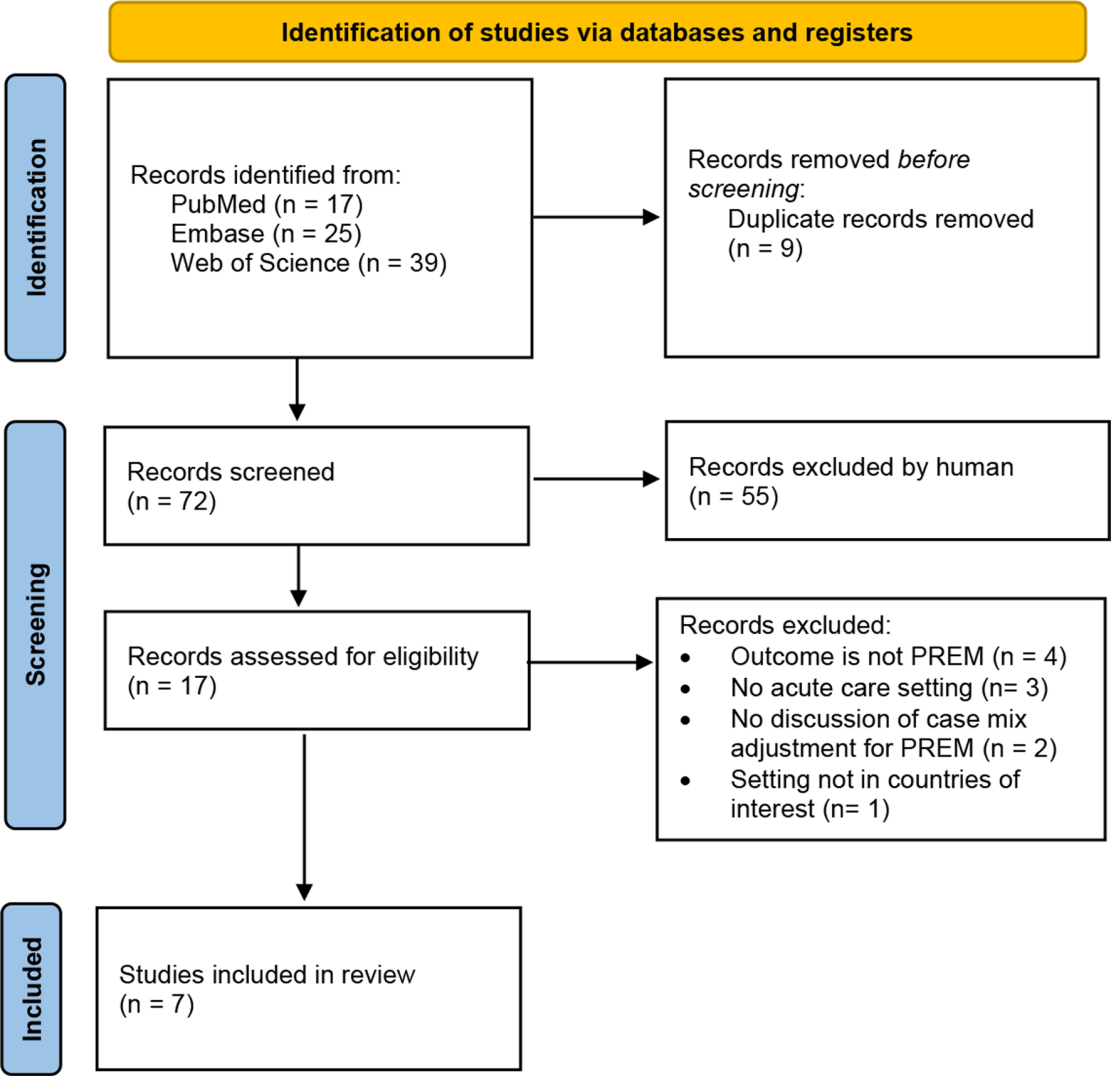
Inclusion flowchart for the identification of studies

The Rapid Review yielded 7 relevant articles. Figure 2 depicts on which grounds the studies were retained. A summary of included articles is described in Table 1. There were 4 studies with data from England, 2 from Norway, 1 from the Netherlands. Sample sizes ranged from 840 to 101’771 patients and between 16 to 280 health centers for which case-mix adjustment was used. Three of the studies from England were based on the same dataset but compared different groups [9], comparing individual trusts [10] comparing trusts in London versus outside London [11] comparing groups of trusts with commond response rates]. There were three studies on cancer patients [9–11], one with dialysis patients [10], two for mental health and substance use patients [12, 13], and one for general inpatient care [14].

**Table 1:** Study characteristics

Study	Setting	N (Patient)	N [Health Centers)	PREM
[9]	Acute hospitals in NHS [United Kingdom]	69,086	160	Cancer Patient Experience Survey
[13]	Secondary care psychiatric institutions (Norway]	1,683	280	Psychiatric Inpatient Patient Experience Questionnaire on site [PIPEQ-OS)
[14]	Substance dependence inpatient care (Norway]	1,452	21	Patient Experience Questionnaire for Interdisciplinary Treaeamtne for Substance Dependence [PEQ-ITSD) 51 questions
[15]	Acute hospitals in NHS [United Kingdom]	57,915	142	Subset of NHS inpatient and A&E patient survey data questions
[10]	Acute hospitals in NHS [United Kingdom]	101,771	158	Cancer Patient Experience Survey
[11]	Acute hospitals in NHS [United Kingdom]	69,086	160	Cancer Patient Experience Survey
[12]	Acute Diabetic Centers in the Netherlands	840	16	Consumer Quality Index

### Selection of case-mix variables

A priori selection of potential case-mix variables was conducted by authors of the included studies based on theoretical considerations, conceptual frameworks, or published literature on the topic. The list of case mix variables included in each study are outlined in Table 2. All 7 studies included age and gender to in the list of case-mix variables for adjustment, 5 for ethnicity, 2 for education, 1 for socioeconomic status, 1 for language spoken, and all included clinical factors such as diagnosis, self-reported health status, or more. One study [14] found that the list of case-mix variables which were deemed appropriate for adjustment differed between the various subscales or dimensions of PREMs.

**Table 2:** Case-mix variables assessed in each study and whether they are recommended for case mix adjustment

Study	Age	Gender	Ethnic Group	Civil Status	Education	Other	Clinical Factors
[9]	x	x	x				Cancer Diagnosis
[13]	x	x		x			Self perceived mental health before admissionOverall current stateDuration of stayType of admissionclinicial understanding situtation, treamtnet adjusted to your situation, adequate information about meantal health condition
[14]	x	x		x	x		self perceived physical and mental healthmost frequently used drug [alcohol)Length of staynumber of previous admissionseage when developed substance dependencemixed substance use
[15]	x	x	x				Admission typePresence of long term conditionProxy responsePrevious A&E attendance
[10]	x	x	x				Cancer Diagnosis
[11]	x	x	x			Socioeconomic deprivation	Cancer Diagnosis
[12]	x	x	x		x	language spoken at home	self-rated healthdiabetes mellitus as primary renal disease (yes; no]; past diagnosis of malignancies (yes; no); past myocardial infarction (yes; no); haemoglobin value (grams per decilitre);serum albumin value (grams per decilitre); status on the transplant (Tx) waiting list (registered; not registered).

### Statistical methods used to assess the impact of case-mix adjustment

All studies included regression models to adjust for case mix, however certain studies used bivariate analysis to determine the list of case mix to be included in the final model [12–14] while others decided to include all a priori case mix variables [9–11, 15].

The majority of studies compared unadjusted and case-mix adjusted regression model to understand the influence of potential case-mix variables on PREM scores. Conveying the impact of case-mix adjustment on PREM however varied across studies. Two studies [9, 15] compared ranks of health centre PREMs scores before and after adjustment to assess the impact of case-mix adjustment. They used Kendall’s Tau to assess rank concordance. Variance explained by case-mix was used by [9], and [11]. Table 3 summarises the risk adjustment method and how the effect of risk adjustment was evaluated.

**Table 3:** Statistical methods used in each study for case mix adjustment

Study	Risk Adjustment Method	Evaluation of Risk Adjustment Effect
[9]	Logistic regression models with a priori case-mix variables [age, gender, ethnic group, and cancer diagnosis)	Degree of reclassification in ranking from bottom 20%, middle 60%, top 20% using Kendall’s Tau. Variance explained by case-mix using logistic regression.
[13]	Bivariate analyses to identify significant case-mix variables, followed by multilevel regression to adjust for case-mix	No comparison between centers. No evaluation of crude versus risk-adjusted PREM scores.
[14]	Bivariate analyses to identify significant case-mix variables, followed by multilevel regression to adjust for case-mix	No evaluation of crude versus risk-adjusted PREM scores.
[15]	Direct standardization and linear regression models with a priori case-mix variables	Proportional reduction in inter-trust variance. Changes in trust rankings using Kendall’s Tau. Performance band shifts.
[10]	Multilevel linear regression to adjust for a priori case-mix variables (age, gender, ethnic group, cancer diagnosis] to compare PREMs between London versus outside London health centers	Comparison between two regions rather than health centers. Graphical presentation of crude versus adjusted PREM Odds Ratios [London vs. Rest of England) by PREM question.
[11]	Multilevel linear regression, adjusting for a priori case-mix variables to compare low, medium, and high response rate groups	No comparison between health centers. Assessment of variance explained by case-mix using logistic regression.
[12]	Univariate models investigate unadjusted relationship with PREM. Subsequent multivariable model constructed using significant variables identified in univariate models.	No comparison between centers, therefore no evaluation of risk adjustment on performance ranking.

### Reporting

Across the reviewed studies, case-mix adjustment was generally recommended to ensure fair comparisons of patient-reported experience measures (PREMs), particularly when results are used for public reporting or performance assessment. While the degree of impact varied, several studies highlighted that adjustment can meaningfully alter performance comparisons. The recommendations from each study are summarized in Table 4.

**Table 4:** Main recommendations on case mix adjustment

Study	Case-Mix Adjustment Recommended?	Parallel Reporting Recommended?	Impact of Case-Mix Adjustment on Performance Evaluation
[9]	Yes	Yes	Case mix has only small impact • High concordance between crude and adjusted. Kendall’s Tau = 0.84 [IQR 0.82–0.88). • 5% of health centers moved out of the extreme performance categories after case mix adjustment.
[13]	Partially	Not discussed	No discussion of crude versus risk-adjusted PREM scores.
[14]	Yes	Not discussed	No discussion of crude versus risk-adjusted PREM scores.
[15]	Yes	Yes	Case mix has small to moderate impact • Inter-trust variance greatest for unadjusted scores, lowest for scores deribed from full regression. • High concordance between three models [Kendall’s Tau = 0.70–0.94] • 14% trusts had disconcordant ranks when using regression model
[10]	Yes	Yes	Case mix has important impact. • Adjusted OR [London vs England]: decreased substantially across questions, e.g., crude OR = 0.68, adjusted OR = 0.83
[11]	Yes	Not explicitly	Patient case mix explained 58% differences between hospitals
[12]	Yes (limited]	Yes	Case mix has only small impact • Minor performance scores; adjustment had limited effect, but recommended for completeness

All studies support using case-mix adjustment to some degree to improve fairness in comparing provider performance. Despite two studies indicating that case-mix adjustment had minimal importance [9, 10], they nevertheless recommend case mix adjustment on the grounds of promoting fairness. And despite recommendation of adjustment of case-mix variables, two studies [11, 12] also cautioned that it risks adjusting away differences that may in fact identify poor quality of care [9, 10, 15] and [12], recommended the simultaneous reporting of both unadjusted and case-mix adjusted scores of health centre performance through PREMs, which can promote transparency of adjustment effects and help to dispel any perceptions of unfair comparisons between health centres serving different patient populations. The degree of impact case mix adjustment had on PREM score ranged from minimal to important.

Overall, the consensus supports the use of a priori case-mix variables and the reporting of both adjusted and unadjusted scores in parallel, ensuring stakeholders can interpret observed differences in light of patient characteristics rather than provider performance alone.

## Discussion

There exists an important ethical dilemma in case-mix adjustment of performance evaluation of health centres. Some suggest that performance indicators should not be case mix adjusted because it disregards the imperative to provide the best possible care to all patients. Otherwise said, case-mix adjustment may be perceived as a permission to provide certain groups with poorer care [16]. However, health centres with higher proportions of patients more likely to report negative experience may be disadvantaged when it comes to performance incentives, which can lead to loss of income, damage staff morale, and undermine the ability to recruit and retain high calibre healthcare professions or attract and retain patients.

The aim of this rapid review was to examine current practices and recommendations for applying case-mix adjustment in the benchmarking of Patient Reported Experience Measures (PREMs) across inpatient healthcare centers. All seven studies reviewed recommended using case-mix adjustment to some extent. However, the list of case-mix variables varied between studies. While some studies concluded that case-mix adjustment had minimal impact on PREM scores [9, 12, 15], and others did not directly assess the effect of case-mix adjustment on PREM scores compared to crude scores [13, 14], case-mix adjustment was still widely recommended.

The benefits of case mix adjustment are to account for systematic differences in patient populations—such as age, gender, health status, and type of admission—which are known to influence reported experiences independently of care quality [9, 14, 15]. Studies have also shown that adjusting for these factors can alter hospital or trust rankings, suggesting that unadjusted comparisons may misrepresent performance [9, 15]. Finally, adjustment is considered essential for equitable and transparent public reporting and accountability, as it prevents unfair disadvantage to providers serving more complex or underserved populations [10, 12].

The main concern from several authors was the risk of masking poor quality of care through case-mix adjustment [9, 10, 12, 15] and therefore should be conducted with caution. One approach discussed by these authors to address this concern was to report both crude and adjusted scores in parallel. Another proposal by [17], which goes beyond the discussions of included studies, proposes a stepwise approach to assess the appropriateness of case-mix adjustment in each situation.

Groenewegen et al. [17] detailed a 3-step approach to assess the suitability of each potential case-mix variable while mitigating the dangers of inappropriate case-mix adjustment. The approach helps determine whether a case-mix variable should be included for adjustment or whether it should rather be assessed for differences that could explain poor quality of care, which is unique to that study. In addition to assessing whether a case-mix variable influences PREM scores, the question whether a case-mix variable was appropriately adjusted for was also posed. The steps include: (1) Test whether each potential case-mix variable influences the PREM score. Use multilevel models with health centres as random effects and one case-mix variable at a time as fixed effects. If there’s no significant effect, adjustment is unnecessary. This approach is used across some reviewed studies [12–14]. (2) Assess whether the relationship (slope) between the case-mix variable and PREM varies significantly across centres. Use multilevel models with random slopes. If slope variance is large (exceeding 25% of total variance), it may reflect true performance differences, and adjustment may not be appropriate. (3) Check whether the case-mix variable is unevenly distributed across centres. Model each case-mix variable as an outcome in a null model. Significant between-centre variation indicates the need for adjustment. If the three criteria apply to the potential case-mix adjuster, then it qualifies according to Groenewegen and colleagues [17] to be a suitable case-mix adjuster.

### Relevance for the Swiss context

The findings from the included studies have important implications for how patient-reported experience measures (PREMs) should be interpreted and reported in Switzerland. While most studies support case-mix adjustment to ensure fair comparisons across health care providers, the specific variables used for adjustment must be contextually appropriate. A key observation is that language spoken was included as a case-mix variable in only one study [10], despite its potential significance. In multilingual Switzerland, language region [e.g., German-speaking vs. French-speaking areas) has been shown to correlate with differences in unadjusted PREM scores. These disparities could reflect cultural differences in reporting style or true differences in care quality, making language a potentially crucial case-mix variable. However, ethnicity, often used in international studies as a proxy for cultural or communicative differences, is not currently captured in Swiss PREMs, and may not adequately replace language in this context. This gap raises concerns about the appropriateness of existing adjustment models if directly applied in Switzerland.

The implication is clear: Switzerland should not adopt case mix strategies wholesale from other systems without evaluation. Instead, following the approach recommended by [17], it would be prudent to first assess whether specific case-mix variables (such as language region] truly affect PREM scores and vary systematically across providers. This staged approach ensures that adjustment is evidence-based and meaningful. Additionally, given concerns across studies that case-mix adjustment can mask true performance differences, parallel reporting of both crude and adjusted results—as recommended by several authors (e.g., Abel et al., Raleigh et al.)—should be strongly considered. This allows for transparency and helps mitigate the risk of adjustment inadvertently legitimizing poorer care for certain groups.

### Strengths & limitations

This Rapid Review is to the best of our knowledge the first summary of evidence on case-mix adjustment for the purpose of PREMs benchmarking. The results will support decision making towards fair benchmarking practices between health centres in Switzerland and potentially also in Europe. A rapid review offers a swift evidence synthesis for addressing urgent questions and providing timely insights for decision-making. They are cost-effective and flexible, allowing researchers to tailor the review process to specific needs and focus on key questions or outcomes. Rapid Reviews also prioritize up–to-date evidence, incorporating recent studies and grey literature to capture emerging trends.

Rapid Reviews come with inherent limitations. Due to the search constraints to ensure a rapid synthesis, we may have omitted informative studies in the process. Rapid reviews may sacrifice depth for speed, offering less detailed analysis and synthesis of findings compared to traditional systematic reviews. Furthermore, the generalisability of the findings to the Swiss context is limited. Although our inclusion criteria allowed for studies across European countries, only studies from three countries (the United Kingdom, Norway, and the Netherlands) ultimately met the inclusion criteria. Among these, there was a disproportionate focus on the UK, particularly studies using NHS data. As such, the case-mix variables, healthcare system structures, and patient populations represented may not fully reflect the diversity or linguistic complexity of the Swiss healthcare system.

## Conclusion

The rapid review aimed to comprehensively analyse current practices and recommendations concerning case-mix adjustment for benchmarking Patient Reported Experience Measures (PREMs) across health centres, with a particular focus on the Swiss healthcare landscape. Among the 7 studies evaluated, unanimous endorsement for case-mix adjustment was observed; however, the compilation of suitable case-mix variables for adjustment varied across studies, presenting challenges in standardization efforts. While acknowledging the potential of case-mix adjustment to facilitate equitable comparisons and pinpoint areas for enhancement, it also raised concerns about its potential to obscure variations in quality across health centres.

Rather than a standardized list of case-mix variables for adjustment in comparing PREMs across health centres, a strategic stepwise approach is recommended to evaluate the appropriateness of case-mix variables, aiming to mitigate the risk of inadvertently masking indicators of suboptimal performance. Emphasizing transparency, it is recommended to disclose both adjusted and unadjusted PREM scores, fostering accountability and ensuring stakeholders have access to comprehensive data. Moreover, the identification and assessment of pertinent case-mix variables, delineated separately for each PREM dimension and context, emerged as imperative considerations. The review highlights the importance of careful consideration and standardized approaches in implementing case-mix adjustment for PREM benchmarking, with particular consideration for implementation in the Swiss healthcare context.

## Data Availability

Not applicable

## Appendix: Search Terms

**PubMed**: (compar* OR benchmark*) AND (((((case mix adj*[Title/Abstract]) OR (risk adj*[Title/Abstract])) OR (adjustment, case mix[MeSH Terms])) AND ((((patient reported outcome measure[MeSH Terms]) AND (experience[Title/Abstract])) OR ((PREM[Title/Abstract]) OR (PREMs[Title/Abstract]))) OR („patient experience”[Title/Abstract:~3]))) AND ((“united kingdom”[MeSH Terms] OR “switzerland”[MeSH Terms] OR “sweden”[MeSH Terms] OR “poland”[MeSH Terms] OR “norway”[MeSH Terms] OR “netherlands”[MeSH Terms] OR “luxembourg”[MeSH Terms] OR “ireland”[MeSH Terms] OR “germany”[MeSH Terms] OR “france”[MeSH Terms] OR “denmark”[MeSH Terms] OR “czech republic”[MeSH Terms] OR “austria”[MeSH Terms] OR UK[Title/Abstract] OR “united kingdom”[Title/Abstract] OR english[Title/Abstract] OR england[Title/Abstract] OR wales[Title/Abstract] OR scotland[Title/Abstract] OR scottish[Title/Abstract] OR welsh[Title/Abstract] OR ireland[Title/Abstract] OR swiss[Title/Abstract] OR swedish[Title/Abstract] OR polish[Title/Abstract] OR norweg*[Title/Abstract] OR netherlands[Title/Abstract] OR dutch[Title/Abstract] OR luxembourg*[Title/Abstract] OR irish[Title/Abstract] OR german*[Title/Abstract] OR french[Title/Abstract] OR danish[Title/Abstract] OR czech*[Title/Abstract] OR austria*[Title/Abstract]))

**Web of Science:** (“case mix” NEAR/3 adjustment OR “risk assessment” OR risk NEAR/3 adjustment) AND (“patient reported experience measure” OR “patient experience” OR “prem” OR “PREMs” OR “patient” NEAR/3 “experience”) AND (“austria*” OR “czech*” OR “czech republic” OR “denmark” OR “danish” OR “france” OR “french” OR “german*” OR “germany” OR “ireland” OR “irish” OR “luxembourg” OR “luxemb*” OR “netherlands” OR “dutch” OR “norweg*” OR “norway” OR “polish” OR “poland” OR “sweden” OR “swedish” OR “switzerland” OR “swiss” OR “united kingdom” OR “england” OR “english” OR “wales” OR “welsh” OR “scotland” OR “scottish”) AND (compar* OR benchmark*)

**Embase:** (((‘benchmarking’/exp OR ‘benchmarking’ OR compar* OR benchmark* OR differenc*) AND ((‘case mix’ NEAR/3 adjusment):ab,ti) OR ‘risk assessment’:ab,ti OR ((risk NEAR/3 ‘adjustment’):ab,ti)) AND ‘patient reported experience measure’:ab,ti OR ‘patient experience’:ab,ti OR ‘prem’:ab,ti OR ‘prems’:ab,ti OR ((‘patient’ NEAR/3 ‘experience’):ab,ti)) AND (‘austria*’:ab,ti AND ‘austria’/exp OR ‘czech*’:ab,ti OR czeck) AND republic OR ‘denmark’:ab,ti OR danish:ab,ti OR ‘denmark’/exp OR ‘france’:ab,ti OR french:ab,ti OR ‘france’/exp OR ‘german*’:ab,ti OR ‘germany’/exp OR ‘ireland’:ab,ti OR irish:ab,ti OR ‘ireland’/exp OR ‘luxembourg’:ab,ti OR luxemb*:ab,ti OR ‘luxembourg’/exp OR ‘netherlands’:ab,ti OR dutch:ab,ti OR ‘netherlands’/exp OR norweg*:ab,ti OR ‘norway’:ab,ti OR ‘norway’/exp OR ‘polish’:ab,ti OR ‘poland’:ab,ti OR ‘poland’/exp OR ‘sweden’:ab,ti OR ‘swedish’:ab,ti OR ‘sweden’/exp OR ‘switzerland’:ab,ti OR swiss:ab,ti OR ‘switzerland’/exp OR ‘united kingdom’:ab,ti OR ‘united kingdom’/exp OR ‘england’:ab,ti OR ‘english’:ab,ti OR ‘england’/exp OR ‘wales’:ab,ti OR ‘welsh’:ab,ti OR ‘scotland’:ab,ti OR ‘scottish’:ab,ti OR ‘scotland’/exp

## Abbreviations

PREM: Patient Reported Experience Measure
